# 3D and 4D Printing of Polymers for Tissue Engineering Applications

**DOI:** 10.3389/fbioe.2019.00164

**Published:** 2019-07-09

**Authors:** Dilara Goksu Tamay, Tugba Dursun Usal, Ayse Selcen Alagoz, Deniz Yucel, Nesrin Hasirci, Vasif Hasirci

**Affiliations:** ^1^BIOMATEN, Center of Excellence in Biomaterials and Tissue Engineering, Middle East Technical University, Ankara, Turkey; ^2^Department of Biotechnology, Middle East Technical University, Ankara, Turkey; ^3^Department of Biological Sciences, Middle East Technical University, Ankara, Turkey; ^4^Department of Histology and Embryology, School of Medicine, Acıbadem Mehmet Ali Aydinlar University, Istanbul, Turkey; ^5^Department of Biomedical Engineering, Middle East Technical University, Ankara, Turkey; ^6^Department of Chemistry, Middle East Technical University, Ankara, Turkey; ^7^Department of Medical Engineering, School of Engineering, Acıbadem Mehmet Ali Aydinlar University, Istanbul, Turkey

**Keywords:** 3D printing, 4D printing, tissue engineering, smart materials, bioprinting, bioinks, scaffold

## Abstract

Three-dimensional (3D) and Four-dimensional (4D) printing emerged as the next generation of fabrication techniques, spanning across various research areas, such as engineering, chemistry, biology, computer science, and materials science. Three-dimensional printing enables the fabrication of complex forms with high precision, through a layer-by-layer addition of different materials. Use of intelligent materials which change shape or color, produce an electrical current, become bioactive, or perform an intended function in response to an external stimulus, paves the way for the production of dynamic 3D structures, which is now called 4D printing. 3D and 4D printing techniques have great potential in the production of scaffolds to be applied in tissue engineering, especially in constructing patient specific scaffolds. Furthermore, physical and chemical guidance cues can be printed with these methods to improve the extent and rate of targeted tissue regeneration. This review presents a comprehensive survey of 3D and 4D printing methods, and the advantage of their use in tissue regeneration over other scaffold production approaches.

## Introduction

Tissues are dynamic structures constituted by multiple cell types, an extracellular matrix (ECM) and a variety of signaling molecules. The extracellular matrix (ECM) is a crucial component of the cellular microenvironment and forms a complex three-dimensional network (Marchand et al., 2018). ECM, with various architectural forms and compositions in different tissues, is a complex 3D network consisting of mainly collagen and elastic fibers, which also contain proteoglycans, multiadhesive proteins (e.g., fibronectin, laminin), and glycosaminoglycans (e.g., hyaluronan). ECM structurally supports and helps the spatial organization of tissues and also serves as the site for cell anchorage. In addition, ECM is a dynamic system that transmits biochemical and mechanical signals from the microenvironment into the cells and affects cell behavior. The development of tissue specific scaffolds that possess the complex hierarchy of natural tissues remains deficient in tissue engineering applications. Three-dimensional printing (additive manufacturing) is achieved by adding materials layer by layer to form the final shape and is a valuable tool in the fabrication of biomimetic scaffolds with desired properties and well-controlled spatial chemistry and architecture. Three-dimensional printing mainly involves the use of 3D software to establish a model; the model is imported into slicing software, and a 3D printer is used to print the model (Bhushan and Caspers, 2017). These 3D constructs, with microporous structures, can be produced through a computer controlled, layer-by-layer process. The conventional production of scaffolds in a sponge or mesh form are achieved by lyophilization, salt leaching, wet spinning and electrospinning. However, it is difficult to obtain pre-determined, well-defined architectures in a controlled manner using these techniques. In addition, cells are seeded onto these scaffolds after fabrication and may not penetrate the depths of the structure; therefore, cells may not be homogeneously distributed within the scaffold. Three-dimensional printing technology overcomes these limitations of conventional scaffold fabrication techniques. The main advantage of 3D printing is the production of patient-specific scaffolds. Four-dimensional printing is an emerging field in tissue engineering, where the scaffolds are fabricated using smart materials that enable the scaffolds to mimic the dynamic nature of tissues to a very large extent. Thus, besides having the advantages of 3D printing, such as the production of scaffolds with well-defined internal organization, 4D printing benefits from the property of smart materials to closely imitate the dynamic responses of tissues against natural stimuli. Four-dimensional printing is an invasive and robust technique that enables users to design the modeled simple shapes to transform to complex designs over time through a programming phase which is distinctly different than 3D printing (Rastogi and Kandasubramanian, 2019). The smart materials used to make the 4D scaffolds respond to a range of stimuli and adapt to the microenvironment by changing their conformation or other properties. The details of 3D and 4D scaffold preparation techniques and the types of stimuli they respond to are presented in this review.

## 3D Printing

Three-dimensional (3D) printing, also known as additive manufacturing or rapid prototyping, plays an important role in tissue engineering applications where the goal is to produce scaffolds to repair or replace damaged tissues and organs. Three-dimensional printing uses a bottom-up approach. Production is guided by a computer model which uses cross-sectional data obtained by slicing magnetic resonance (MR) or digital image of the defect area. Thus, production in a layer-by-layer fashion is possible using this technique, with high structural complexity, especially for patient-specific implants (Peltola et al., 2008). The main 3D printing categories that use solid polymers for product formation are; fused deposition modeling (FDM), selective laser sintering (SLS), and stereolithography (SLA). Bioprinting which uses polymeric hydrogels loaded with cells constitutes another category. The principles of these techniques are presented below.

### 3D Printing Techniques Using Polymers

#### Fused Deposition Modeling (FDM)

**Figure 1:**
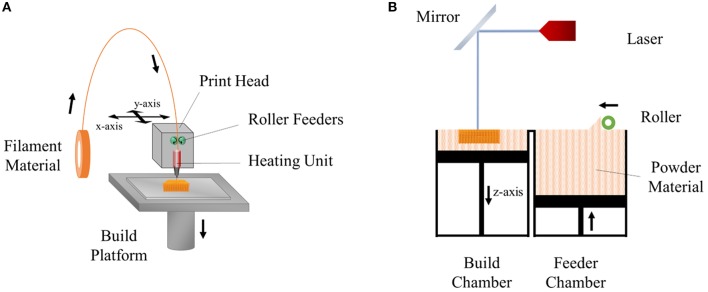
Schemes of **(A)** fused deposition modeling (FDM) and **(B)** selective laser sintering (SLS) techniques.

Fused deposition modeling (FDM) was developed and patented by Scott Crump in the late 1980s and is one of the most commonly used rapid prototyping techniques. This technology has been used in a broad range of applications including automotive, aerospace, model production for visualization, design verification, and biomedicine (Casavola et al., 2016). FDM is based on heating a thermoplastic polymer introduced to the device (in the form of a filament or powder), in the heating chamber, to a molten state which is then extruded through a nozzle onto the platform where it is deposited layer-by-layer in order to construct a 3D form. During the fabrication process, the position of the nozzle is controlled by a computer program and moves in x-y plane in order to create the desired pattern. Once a layer is completed, the nozzle moves upwards along the z-axis, a predefined distance to print the next layer. This process continues until the desired form is created (Xu et al., 2014). The components of FDM are shown in Figure 1A. The resolution of the details of the product is defined by the nozzle diameter, print speed, the angle and the distance between fibers of the subsequent layers, and the number of layers (Yuan et al., 2017).

The key advantage of FDM is the possibility of multiple extrusions with different materials. In the process, nozzles containing different thermoplastic materials are controlled by the system where they extrude sequentially, and the total form composed of the varied properties can be obtained. Other advantages of the FDM are simplicity, cost effectiveness and high speed (Wang et al., 2017). This method is solvent-free, therefore, an organic solvent (e.g., chloroform, acetone) which may be toxic or damaging for the cells is avoided (Thavornyutikarn et al., 2014). The disadvantage of the technique is the limited number of usable thermoplastic materials; as medical grade, biocompatible materials are not abundant. Additionally, it is difficult to find materials with the proper melt viscosity, which should be high enough to deposit and low enough to extrude (Chia and Wu, 2015).

#### Selective Laser Sintering (SLS)

Selective laser sintering (SLS) is an additive manufacturing (AM) technique which was developed and patented by Carl Deckard and Joe Beaman in 1989 (Deckard, 1989). In this technique, a laser beam is used as the energy source which melts a thin layer of powder material (ceramics, metals, and thermoplastic polymers) spread in the form of a powder bed. The beam heats the material and fuses them together to draw the 2D shape according to the computer program. After a layer is produced, the built platform moves down one-layer of thickness, and a new layer of powder is spread on the surface of the platform by a piston to sinter on the next layer. This process is repeated until the final structure is built (Mazzoli et al., 2015) (Figure 1B). After the fabrication is completed, excess powder is removed either by brushing or application of compressed air (Mazzoli, 2013).

SLS offers the advantage of fabricating large and complex scaffolds. Another advantage is that SLS does not require any support structures during the production process, since the sintered object is located in a solid powder bed and a sacrificial layer is not needed (Bai et al., 2015). SLS is a solvent-free fabrication method (like the FDM) thus the printed product does not have traces of the remaining solvent. The main disadvantage of SLS is that the product surface is not smooth and needs polishing because the product, and naturally its surface, is created by fusing spherical particles which introduce a certain degree of roughness (Mazzoli, 2013).

#### Stereolithography (SLA)

Stereolithography (SLA) is based on selective polymerization of a liquid, photosensitive resin by a light source, such as UV light or a laser (Mondschein et al., 2017). In the early 1980s, the first study on the fabrication of the 3D structure, through the photopolymerization of the liquid-based resin utilizing UV light, was achieved by Kodama, who developed two approaches, one utilizing a mask for each layer to do the exposure through, and the other using an optical fiber to cure the photopolymer selectively (Kodama, 1981). A predefined design was created by controlling the fiber movement along the x and y axes. Hull (1986) contributed to this by the addition of movement along the z-axis to produce 3D scaffolds in a layer-by-layer approach via UV light (Zorlutuna et al., 2013; Du, 2018).

In essence, stereolithography is a dynamic version of photolithography and uses a narrow beam of light to cure the polymer to produce the desired pattern, unlike photolithography which uses a static photomask to build a micropattern (Cha et al., 2014). In this system, light selectively polymerizes the resin according to a computer aided design (CAD) model. After the formation of the first layer, the platform is lowered, and a fresh resin material is added to polymerize and to create the second layer. It can also be achieved by moving the product in the z-direction after dipping into the liquid medium. Finally, uncrosslinked resin between the layers is washed, the construct is post-cured with UV in order to complete the polymerization reactions and increase the stability of the product (Melchels et al., 2010).

**Figure 2:**
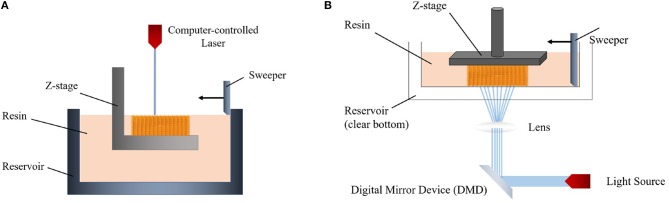
Schemes of **(A)** laser-based stereolithography and **(B)** digital light projection (DLP) system.

In order to cure the resin of the two different irradiation approaches, laser-based stereolithography and digital light projection (DLP) can be used. In the laser-based method, a laser beam which is controlled by a computer directly writes an object in a bottom-up way (Figure 2A) (Skoog et al., 2014). The required light intensity for printing is controlled by a digital micro-mirror device (DMD) which uses microscale mirrors aligned in an array (Figure 2B). Each mirror can be rotated independently in this array to on-and-off states. Thus, only the desired area is exposed to light and polymerized (Lee et al., 2015).

SLA offers many advantages over the other techniques. First, each layer is printed at the same time when multiple objects are being printed, and total printing time is only based on the structure thickness. This significantly decreases printing time (Wang Z. et al., 2015). Also, external geometry and internal architecture of the scaffold can be precisely controlled by SLA due to a high resolution advantage of accuracy at 20 μm, due to the width of the light source being very small and highly controlled (Ji et al., 2018). Thus, the complex scaffold can be easily fabricated. The main disadvantage is that only a few biocompatible materials are available to be used in SLA to produce tissue engineering scaffolds (Colasante et al., 2016).

#### Near-Field Electrospinning (NFES)

**Figure 3:**
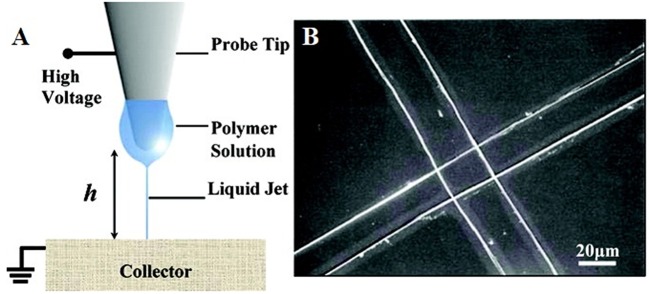
**(A)** Schematic representation of NFES system and **(B)** perpendicular fibers deposited using the NFES system (adapted with permission from Sun et al., 2006. Copyright 2019 American Chemical Society).

Electrospinning (ES), a traditional scaffold production technique frequently adopted in tissue engineering applications, is based on the uniaxial elongation of a viscoelastic jet of a polymer solution or melt under high voltage (Li and Xia, 2004). Although it is an advantageous method of building micro and nano fibers and structures due to its simplicity, efficiency, and variety in applicable materials and fields, it lacks the precision that some areas, such as microelectromechanical systems (MEMS) and tissue engineering require. Near-field electrospinning (NFES), introduced first by Sun et al. (2006), applies the same principle as traditional electrospinning (TES) but with low voltage and reduced working distance to achieve controlled deposition of fibers and precision in the spun structures (Figure 3). With the reduced working distance, bending instability that arises in TES is significantly restrained, so that the fibers can be deposited as straight lines rather than randomized chaotic patterns. The collector, unlike in TES, is placed on a platform that moves along the x and y-axes, and this movement is precisely controlled by a computer program that enables laying fibers down in a predetermined path to obtain a desired pattern or shape in 2D, or 3D by depositing fibers layer by layer. Similar to TES, NFES also work with polymer solutions and melts. Table 1 summarizes the differences between TES and NFES.

**Table 1:** Comparative analysis of traditional electrospinning (TES) and near-field electrospinning (NFES) (adapted with permission from He et al., 2017. Copyright 2019 American Chemical Society).

**Method**	**Forms**	**Working distance (cm)**	**Voltage applied (kV)**	**Collector type**	**Fiber diameter (μm)**	**Advantages**	**Disadvantages**
TES	Solution	5–50	10–30	Static	0.01–1	Device simplicity	Random fiber deposition
	Melt			Dynamic		Variety of usable materials	High voltage
						Large-scale production	
NFES	Solution	0.05–5	0.2–12	Static	0.05–30	Controlled fiber deposition	Immature mechanism
	Melt			Dynamic		Low voltage	Larger fiber diameter
						Precision in structures built	Small-scale production

NFES has some trade-offs between the controllability of fibers and the morphology of the structure (He et al., 2017). The shortened distance between the tip and the collector enables accurate fiber deposition while limiting the stretching and thinning of fibers, resulting in fiber diameters larger than those observed in TES. Some research shows that this issue can be improved by introducing minor modifications in solution concentrations, spinning voltage and distance, and collector speed. Also, in contrast to TES where the polymer solution can be deposited continuously from a syringe pump, NFES requires dipping of a probe tip intermittently into the polymer solution, which hinders the continuous large-scale production of micro and nano fibers.

### Bioprinting

**Figure 4:**
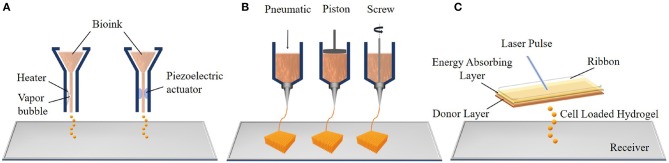
Components of the three main bioprinting techniques: **(A)** inkjet bioprinting, **(B)** extrusion bioprinting, and **(C)** laser assisted bioprinting (LAB).

Bioprinting is another 3D fabrication technique which prints complex tissue constructs using hydrogels that are loaded with cells to print. This technology has the potential to generate a variety of transplantable soft tissues, including skin, bone and cartilage (Mandrycky et al., 2016). Bioprinting has three major process approaches: inkjet, extrusion, and laser-assisted bioprinting (Figure 4) which are described below.

#### Inkjet Bioprinting

The Inkjet was the first bioprinting technology of additive manufacturing and was developed by the Hewlett-Packard Company in the 1970s as a 2D printing method. Then, an elevator platform which can move along the z-axis and a chamber were added to this system in 1992 and a 3D bioprinting system was developed (Huang et al., 2017). Thermal and piezoelectric inkjet bioprinters are more frequently used for tissue engineering applications. In thermal inkjet bioprinting, a prepolymer solution which can contain cells, known as the bioink, is loaded in an ink cartridge. The cartridge is placed in the printer head which is controlled by a computer and small droplets of ink are ejected by the help of small air bubbles created by heat in the printing head. The size of droplets can be changed with ink viscosity, the frequency of the current pulse and the gradient of applied temperature (Cui et al., 2012). The working principle of the piezoelectric inkjet bioprinter is based on applying different potentials to the piezoelectric crystal in the bioprinter, and this generates the pressure needed to eject the bioink droplets from the nozzle. The major advantages of inkjet bioprinting are its fast fabrication and the affordability of the device (Murphy and Atala, 2014).

#### Extrusion Bioprinting

Extrusion bioprinting, an advanced version of inkjet bioprinting, dispenses bioink using pneumatic (air pressure) or mechanical (screw or piston) systems. In the pneumatic system, bioink is extruded from the nozzle or needle as an uninterrupted cylindrical filament by applying continuous air pressure instead of single droplets. This provides high structural integrity to the product (Knowlton et al., 2018). The mechanical system enables a more direct control over the flow of bioink because of the screw extruding the material. Extrusion bioprinting can print tissues using a variety of bioinks, such as cell-carrying hydrogels, micro-carriers and cell aggregates (Ozbolat and Hospodiuk, 2016). However, the cells are subjected to high mechanical stresses during extrusion which may decrease the cell viability (Mandrycky et al., 2016). In addition, the main problems of both inkjet and extrusion bioprinting are clogging of the nozzle due to cell aggregation, high viscosity of the ink or drying of the injected material within the nozzle.

#### Laser-Assisted Bioprinting (LAB)

Laser-assisted bioprinting (LAB) is another bioprinting system which consists of a pulsed laser source, a donor layer, and a receiving substrate. The principle of LAB is that, bioink is placed below a ribbon which also contains a thin, energy absorbing layer. The ribbon is placed parallel to the receiver. The pulsed laser source is focused on the laser absorbing-layer and this generates a vapor bubble. This bubble creates a pressure to deform the bioink and forms droplets. These cell loaded hydrogel droplets are propelled toward the receiver where they are collected and crosslinked (Gruene et al., 2011). LAB offers certain advantages including not clogging due to the absence of a nozzle and not causing any mechanical stress on the cells because of its non-contact printing approach. All of these increase the cell viability. However, the LAB system is more expensive compared to other bioprinters (Mandrycky et al., 2016).

### Materials Used in 3D Printing

A variety of biomaterials are used in additive manufacturing to form the desired, complex-shaped products with different sizes and stiffness. Polymeric materials are generally preferred because of their easy processability, biodegradability, biocompatibility, and low cost. These materials are used in the form of filaments and powders in FDM and SLS, and as bioinks for SLA and bioprinting. In this section, properties of materials used in 3D printing and bioprinting are discussed.

#### Fibrous Materials

Fiber-based thermoplastic polymeric materials are commonly employed in fused deposition modeling (FDM), also known as fused filament fabrication (FFF), and is the simplest 3D printing method. Polymeric filaments must have a certain diameter to fit the heating and extruding head of the printer. Quite a number of commercial filaments, such as acrylonitrile butadiene styrene (ABS), and poly(lactic acid) (PLA) are available on the market. ABS is the most preferred 3D printing material for FDM applications because of its relatively low glass transition temperature and absence of crystallites due to it being an amorphous polymer. These properties enhance accuracy in printing and dimensional stability of the product because the shrinkage ratio during the cooling step is very small. However, the use of ABS filaments in tissue engineering is limited due to its non-biodegradable and non-biocompatible nature (Rosenzweig et al., 2015). A PLA filament is another material used because of properties similar to that of ABS. PLA is an environmentally friendly material that can be produced using natural sources, such as beets and corn. Its biodegradability and biocompatibility make it a good alternative to petroleum-based materials like ABS. It can generally print at temperatures between 200 and 230°C (Guvendiren et al., 2016).

A major disadvantage of the commercial filaments is that their composition is unknown. Therefore, their biocompatibility is not certain and medical use is not possible (Ravi et al., 2017). Extensive cytotoxicity and other biocompatibility tests are needed for these materials prior to any biomedical application. To overcome the biocompatibility problem and unknown ingredients, some researchers fabricated filaments from PCL and PLA pellets using an extruder (Hutmacher et al., 2001; Senatov et al., 2016a).

#### Powder Materials

The majority of the powder-based materials used in additive manufacturing systems are polymers. The techniques generally applied are fused deposition modeling (FDM) and selective laser sintering (SLS).

Poly(caprolactone) (PCL) is a common polymeric material utilized by FDM and SLS for tissue engineering applications because of its low melting temperature (55–60°C), excellent viscoelastic and rheological properties in addition to its a biodegradability and biocompatibility. It is approved by the FDA (Food and Drug Administration of USA) for certain medical applications (Brunello et al., 2016). It is also a stable material in the human body; it can stay for more than 6 months without significant degradation and its complete degradation could take around 2 years. However, the molecular weight, form, porosity and surface area of the material can change this duration significantly. The degradation profile and high stiffness of PCL make it a good molecule particularly for bone tissue engineering. PCL blended with hydroxyapatite (HAP) and tricalcium phosphate (TCP) is also used as printing material for bone tissue engineering where HAP serves to promote osteoinductive and osteoconductive properties of the printed scaffolds (Eosoly et al., 2010; Park et al., 2011; Mota et al., 2015).

Poly(D,L-lactic acid-co-glycolic acid) is one of several commonly used PLGA copolymers and is another synthetic thermoplastic polymer approved by the FDA for clinical use. It was used in FDM applications to fabricate scaffolds due to its processability and high mechanical strength (Do et al., 2015). Nevertheless, the high glass transition temperature of PLGA necessitates high temperatures to create the required flow viscosity for extrusion from the nozzle (Maniruzzaman, 2019).

The powders of block copolymers of polyethylene oxide terephthalate (PEOT) and polybutylene terephthalate (PBT), are thermoplastic elastomers employed in FDM applications. These block copolymers display excellent properties, such as toughness and elasticity, biocompatibility and easy processability. However, PEOT/PBT has been less studied compared to PCL and PLA because it requires an extremely high melting point (225°C) (Moroni et al., 2005).

Polyether ether ketone (PEEK) is a semi-crystalline thermoplastic polymer which has extensively been utilized in SLS. PEEK has a high elastic modulus similar to cortical bone, making it a good alternative to metal implants (Mazzoli, 2013). It is also biocompatible, bioinert, and heat resistant but not degradable, thus not suitable for tissue engineering. PEEK can only be processed by SLS technique due to its very high melting point (350°C) (Schmidt et al., 2007). Sintered PEEK and PEEK/HA have been employed for various orthopedic applications (e.g., joints) (Kurtz and Devine, 2007).

Poly(vinyl alcohol) (PVA), and blends of PVA and HAP have been studied as other powder-based materials for SLS for use in cartilage and bone tissue engineering (Chua et al., 2004; Shuai et al., 2013). PVA is a semi crystalline copolymer composed of vinyl alcohol and vinyl acetate units. It is bioinert, biocompatible, biodegradable and can be sintered at a low temperature (65°C) (Wiria et al., 2008).

Types of powder-based polymers used to produce scaffolds by FDM and SLS are summarized in Table 2.

**Table 2:** Summary of commonly used fiber and powder-based polymers for 3D printing and their advantages and disadvantages.

**Polymer**	**State of starting material**	**Technique**	**Advantage**	**Disadvantage**	**References**
ABS	Filament	FDM	Low T_g_	Non-biodegradable	Rosenzweig et al., 2015
			Easy processability	Non-biocompatible	
PLA	Filament	FDM	Flexibility	High melting point (200–230°C)	Guvendiren et al., 2016
			High mechanical properties		
PCL	Powder	FDM, SLS	Low melting temperature (55–60°C)	Slow degradation	Ravi et al., 2017
PCL/HAPaannggeell PCL/TCP			Excellent viscoelastic and rheological properties		Hutmacher et al., 2001; Brunello et al., 2016; Senatov et al., 2016b
PLGA	Powder	FDM	Higher processability and mechanical strength	High T_g_	Eosoly et al., 2010; Park et al., 2011
PEOT/PBT	Powder	FDM	High toughness and elasticity	High melting point (225°C)	Mota et al., 2015
			Easy processability		
PEEK	Powder	SLS	High elastic modulus	High melting point (350°C)	Do et al., 2015; Maniruzzaman, 2019
			Heat resistance		
			Bioinert		
PVA	Powder	SLS	Bioinert	Low mechanical properties	Moroni et al., 2005; Mazzoli, 2013
PVA/HAP					Schmidt et al., 2007

#### Bioinks

Bioinks are the main constituents of bioprinting and stereolithography which are important for the printing of 3D tissues and organs. Bioinks should have certain characteristics to serve as printing materials. They should be biocompatible (not cause any immune or undesirable response after implantation), printable (as printing materials), and robust (resist physical forces of the environment) (Mosadegh et al., 2015). Today, hydrogels are the most commonly used bioinks and they can easily be loaded with cells. They are preferable because of their printable, cross-linkable, biocompatible nature and high swelling capacity. Hydrogel sources can be natural or synthetic (Mandrycky et al., 2016).

Natural hydrogels are mainly polysaccharides (e.g., chitosan, alginate, agarose) and components of the extracellular matrix (ECM) (e.g., collagen, gelatin, fibronectin, and laminin) (Zorlutuna et al., 2013). Alginate is a natural linear polysaccharide obtained from the wall of brown algae. It is widely used in 3D bioprinting applications due to its biocompatibility, promotion of cell proliferation, low price and the ability of fast gelation in calcium ion containing solutions. The major limitation of alginate derived hydrogels is mechanical stiffness for 3D bioprinting (Du, 2018). Agarose is another linear polysaccharide which is in gel form at room temperature when hydrated, but it can revert to solution form when the temperature is raised above 37°C. Chitin is a major constituent of crustaceous animals and chitosan is a linear polysaccharide that is obtained by deacetylation of chitin. However, it is not suitable to print large scale scaffolds due to its low mechanical strength and low gelation speed. Gelatin and collagen are highly biocompatible materials and enhance cell proliferation. The methacrylated form of gelatin (GelMA) can be easily printed by bioprinters and then stabilized by exposure to UV irradiation (Zhang X.-F. et al., 2017).

Synthetic hydrogels are produced chemically in the laboratory; thus, their mechanical and chemical properties can be controlled by the route or the conditions of the preparation process. Photosensitive synthetic hydrogels, such as polyethylene glycol diacrylate (PEGDA) are generally used as resins in stereolithography (SLA) (Du, 2018). PEG is chemically modified with acrylate groups to form the photopolymerizable polyethylene glycol diacrylate (PEGDA) in which cells can be entrapped in Skardal and Atala (2015). The major limitation of hydrogels is that the bioprinted structure tends to collapse because of low viscosity and low mechanical strength (Billiet et al., 2012).

Poly(propylene fumarate) (PPF) is also a photo-crosslinkable polymer utilized in stereolithography and overcomes some limitations of synthetic hydrogels, such as lower mechanical strength and a lack of biodegradability. PPF polymer is generally mixed with a photoinitiator and a solvent like diethyl fumarate (DEF). Printability and mechanical properties of the scaffold depend on the PPF/DEF ratio (Lee et al., 2015).

## 4D Printing

Additive manufactured structures using smart (intelligent) materials are able to self-transform into a predefined shape or exert a predefined function depending on the stimuli present in the microenvironment; these processes are regarded as “4D printing” (Tibbits, 2013, 2014; Pei, 2014; Choi et al., 2015). Four-dimensional printing utilizes the same additive manufacturing techniques and devices discussed above in the 3D printing section. What constitutes the main difference between 3D and 4D printing is the nature of the materials used. For a 3D printed product to be considered 4D printed, it should exhibit at least one type of smart behavior, such as “shape memory” or “self-actuation” (Table 3) (Li X. et al., 2016). Four-dimensional printing has several advantages over 3D printing (Table 4). Introduction of the fourth dimension, time, in addition to the 3D arrangement gives both spatial and temporal control over the fabricated product. Therefore, 4D printing overcomes one of the major drawbacks of 3D printing and produces structures that are dynamic and animate. Smart materials are most commonly referred to as “materials that exhibit changes in physical or chemical properties in a controlled and functional manner upon exposure to an external stimulus, such as heat, moisture, light, magnetic field or pH.” Thus, a 4D printed product can change its shape, color, function or other physical or chemical properties in response to the aforementioned stimuli types. Programmability of the state and function of the 4D printed product as a result of the smartness of the material eliminates the need for external devices or methods for post-processing, and reduce the production duration, and in some cases may also aid in the application process (Tibbits, 2014). For example, shape changing smart scaffolds that exhibit compactness prior to *in vivo* application could be used in minimally invasive procedures and self-assembly to the required complex shape due to dynamic response upon implantation (Miao et al., 2016a).

**Table 3:** Types of smart behavior observed in responsive materials.

**Smart behavior**	**Description**	**Examples**	**References**
Shape memory	Material changes into a predefined shape in response to an external stimulus	Poly(ε-caprolactone) dimethacrylateaannggeell (PCLDMA)	Neuss et al., 2009
		Poly(ether urethane)	Cui et al., 2011
		Polyimide	Zhang and Ionov, 2014
Self-assembly	Exposure to external stimulus induces folding of chains and assembly into a preprogrammed shape	4,4′-diglycidyloxyazobenzene polymerized with sebacic acid	Li Y. et al., 2016
Self-actuating	Automated actuation of material upon exposure to an external stimulus	*N*-isopropylacrylamide (NIPAM) and ruthenium(II) tris-(2,2′-bipyridine) copolymer	Tabata et al., 2002
Self-sensing	Material detects and quantifies the exerted external stimuli	Mechanophore crosslinked poly(methyl acrylate) and poly(methyl methacrylate)	Davis et al., 2009
Self-healing	Damage caused in the structure is repaired without any external intervention	Microencapsulated dicyclopentadiene (DCPD)-Grubbs' catalyst embedded in epoxy matrix	White et al., 2001
		Poly(ethylene-co-methacrylic acid) copolymers and ionomers	Kalista and Ward, 2006; Kalista et al., 2007

**Table 4:** Comparison of 3D printing and 4D printing.

**Property**	**3D printing**	**4D printing**
Manufacturing process	2D sections of a 3D structure (with respect to the z-axis) are built layer-by-layer from top to bottom or from bottom to top	Produced in the same way as 3D printed products, but changes shape or function after manufacturing, upon exposure to a specific stimulus
Materials used	Thermoplastic polymers, ceramics, metals, biomaterials, and their composites	Smart materials (polymers, ceramics, metals, biomaterials, and composites) that undergo a change in property or function over time in response to a specific stimulus
Material programmability	Not possible	Material properties and functions are programmable with a specific exposure sequence and time of stimulus, and the spatial organization of material in desired final product
Object shape/function	Stable over time	Object shape or function changes over time when structure is exposed to a specific external stimulus
Application areas	Fields including but not limited to medical, engineering, dentistry, automotive, jewelry etc.	All 3D print application areas where a dynamic change in configuration is required or beneficial.

### Factors Influencing 4D Printing

Five main factors influence the process of 4D printing: (i) type of additive manufacturing process, (ii) type of responsive material, (iii) type of stimulus, (iv) interaction mechanism between stimulus and the material, and (v) mathematical modeling of the material transformation.

Additive manufacturing process, as in 3D printing, realizes the spatial geometry provided by the digital information produced in computer aided design/manufacturing (CAD/CAM) programs. Many additive manufacturing processes that are commonly used, such as fused deposition modeling (FDM) (Hendrikson et al., 2017) and stereolithography (SLA) (Miao et al., 2018) are also suitable for 4D printing applications. Stimuli responsive material and additive manufacturing processes should be compatible with each other in 4D printing, as in the material selection process in 3D printing applications. In the case of multi-material structures where the difference in material properties (swelling, thermal expansion, etc.) drive the transformation of the shape or function, the additive manufacturing process selected should support the homogeneous distribution of the material and produce a single printed structure.

The most important element of 4D printing is the responsive material, since it is the material which introduces the fourth dimension into the process. The time-dependent change observed in the responsive material upon exposure to a stimulus can be physical or chemical. Some responses include folding, curling, twisting, expansion, contraction, color change, and degradation (Li Y. C. et al., 2016).

**Figure 5:**
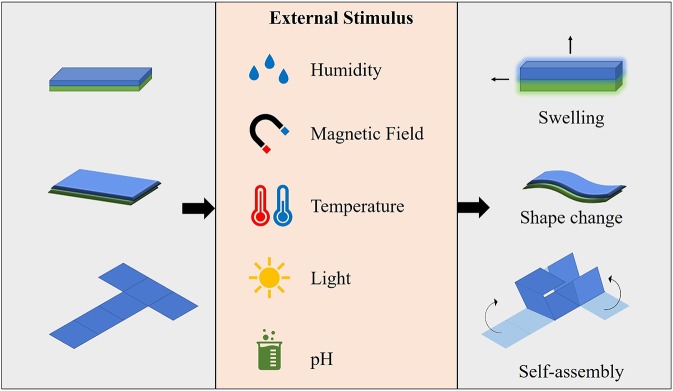
Types of stimuli, and responses observed in smart materials.

The stimulus enforces the transformation of shape or function of the responsive material, thus the 4D printed structure is manipulated over time, after the manufacturing process. Types of stimuli that act on responsive materials can be physical, chemical or biological. Physical stimuli include temperature, light, and magnetic field, whereas humidity, and pH are examples of chemical stimuli (Figure 5). Some smart materials have multi-functionality and respond to two or more signals simultaneously (Zhang et al., 2015).

Types of stimuli to be exerted, and thus the smart material, should be selected with care, taking into consideration the requirements and constraints of the specific application area, and the relevance of the interaction mechanism to the selected application. Interaction mechanism refers to the process of application of stimulus to the responsive material. In some applications, transformations require additional processes prior to application of the stimulus, rather than simple exposure of the material, to obtain a response and the desired outcome. For example, in the case of constrained-thermomechanics, the smart material exhibits a shape memory effect upon being exposed to heat. In order to achieve the shape memory function, however, the material is first subjected to an external load at a high temperature and deformed. Then temperature is lowered while the structure is still under load. The load is removed at low temperature and the material is molded in this predetermined shape. When heat is applied as the stimulus, the earlier form is regained.

In order to achieve a successfully programmed and controlled 4D effect for a specific application, theoretical and numerical models are generally utilized. These models aid in predicting the appropriate exposure sequence of stimuli, and time required for the structure to reflect the desired behavior by establishing connections between material and stimulus properties, structure, and the desired final shape. The targeted 3D spatial orientation and material distribution in structure, and the estimation of system behavior with different material properties and geometries are tested with these models through a finite-element analysis (FEA).

An ongoing debate about 4D printing is whether gradual degradation of 3D printed constructs can be categorized as a time-dependent 4D effect (Choi et al., 2015; Zhou et al., 2015). Scaffolds manufactured from biodegradable biomaterials and the sustained release of therapeutic agents from such scaffolds would have to be included in 4D printing applications if the biodegradation process is a strictly programmable, time-dependent phenomenon. While biodegradability is a desired material property in the field of tissue engineering, an important requirement of the transformation through the 4D process is that the structure must display a minimum of two stable configurations or shapes before and after the triggering stimulus is applied, because the responses to the stimuli should be reversible (Zhou et al., 2015). Thus, 3D structures based on self-degrading polymers are not regarded as having a 4D effect.

### Smart Polymers for 4D Printing in Tissue Engineering Applications

Tissue engineering and regenerative medicine have -to some extent- overcome the challenge of fabricating complex tissue/organ geometries and controlling the tissue microarchitecture with the aid of 3D printing. Recently, tissue mimics and scaffolds that are capable of sensing the dynamic tissue microenvironment and adapting their shape or chemistry are also in increasing demand. Such products fabricated by combining stimuli responsive materials and 3D printing are expected to improve responses to pathology (Morrison et al., 2015), and allow application of minimally invasive surgical procedures (Javaid and Haleem, 2018) and insertion of implants to sites that are otherwise not accessible (Zarek et al., 2016a).

Stimuli responsive polymers, as explained previously, undergo physical or chemical changes when exposed to appropriate stimuli. The cause of this responsiveness is the presence of certain functional groups along the polymer backbone that are sensitive to a change in state, such as charge or polarity. The resulting changes in chemical structure lead to the macroscopic level transformations i.e., changes in chain dimensions and size, secondary structure, solubility, degree of intermolecular association, sol-gel transition, and even chain breakage (Aguilar and San Román, 2014).

An important aspect of some stimuli responsive polymers is the reversibility of the response; meaning that the material is able to return to its original state upon removal of the stimulus. A natural example of this would be the hygroscopic folding and unfolding of pinecone scales, in response to the level of humidity. The scales contract upon increase of humidity and expand when the humidity level is low, scattering the seeds they hold inside (Song et al., 2015). This reversibility of response introduces some drawbacks to the utilization of the said polymers in 4D printing processes; impaired printability of the material, and reproducibility of the desired 4D effect in the product (Lee A. Y. et al., 2017). To overcome these issues, stimuli responsive polymers can be used in combination with other polymers or ceramics where the non-responsive component may serve as a biological or mechanical property enhancer (Senatov et al., 2016b, 2017), or a processing aid.

The mechanisms behind responsiveness to various stimuli exhibited by smart polymers are summarized in this section, along with some examples that have been utilized in 3D printed tissue engineering applications. Responsiveness of polymers are categorized into two classes; responsiveness to (i) physical stimuli and (ii) chemical stimuli. Temperature responsive, photo responsive, and magneto-responsive polymers fall under those that respond to physical stimuli, while pH and humidity responsive polymers are classified under responsiveness to chemical stimuli.

#### Responsiveness to Physical Stimuli

##### Temperature responsive polymers

Temperature responsive polymers are among the most frequently used materials in 4D printing applications especially in the tissue engineering field where a change in temperature can be easily controlled and applied in a non-invasive manner. Many applications exploit the human body temperature, 37°C, to trigger the desired response of some materials. The two most common classes of temperature responsive polymers used in 4D printing applications are (i) shape memory polymers, and (ii) responsive polymer solutions (Hoogenboom, 2014).

**Figure 6:**
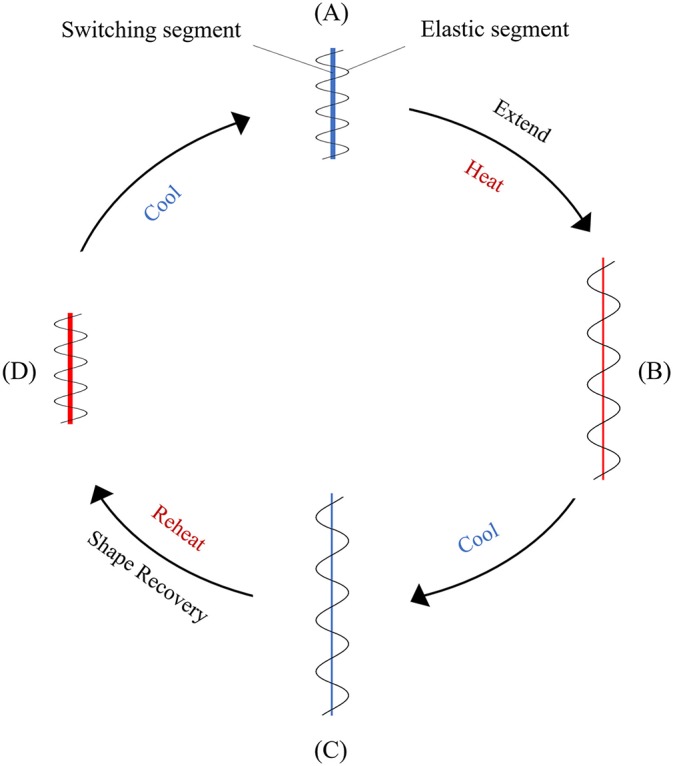
Schematic representation of the mechanism in a temperature responsive SMP. **(A)** Original conformation where the elastic and the switching segments are entangled; **(B)** upon stretching at high temperatures the switching segment becomes soft and is deformed, and the elastic segment is extended; **(C)** when the temperature is lowered, the switching segment hardens, preventing the recovery of the elastic segment; **(D)** after reheating, the original shape is recovered because of the softening of the switching segment and the release of the elastic energy from the pre-deformed elastic segment.

Shape memory polymers (SMP) that have temperature responsiveness are thermoplastic elastomers consisting of two distinct components, one is the elastic segment with a high glass transition temperature (T_g, 1_) and the other is the switching segment with intermediate glass transition temperature (T_g, 2_) or melting temperature (T_m_) (Figure 6). When deformed at a temperature above the highest T_g_, these materials obtain their permanent shape. At a temperature between the two glass transition temperatures, the switching segment becomes soft and pliable while the elastic segment resists the applied constraint, such as stretching or compressing. After deformation at this state, if the polymer is cooled below the glass transition temperature of the switching segment (T_g, 2_), a temporary shape is formed. At this stage the elastic segment cannot return to its original form even after the removal of applied constraints. The driving energy for the shape change effect of the polymer is the elastic spring energy contained within this segment. When the polymer is heated above T_g, 2_ again, the elastic segment is able to drive the shape change effect that transforms the polymer back to the original permanent shape (Sun et al., 2012).

Shape memory polymers have some advantages and drawbacks compared to their metal and ceramic counterparts. The advantages include low density, low cost, ease of shape manipulation and good control over recovery temperature, high strain recovery, and physical and chemical modification ability to achieve desired properties (e.g., biodegradability). As drawbacks compared to metals and ceramics they have lower strength, elastic modulus and processing temperatures.

Some examples of shape memory polymers used in 4D printed tissue engineering applications are poly(ε-caprolactone) dimethacrylate (PCLDMA) (Neuss et al., 2009), soybean oil epoxidized acrylate (Miao et al., 2016b, 2018), polycaprolactone triol (Ptriol) (Miao et al., 2016a), poly(ether urethane) (PEU) (Cui et al., 2011), and poly(lactic acid) (Senatov et al., 2016b, 2017).

Responsive polymer solutions are generally copolymers that have a critical solution temperature that affect the hydrophilic and hydrophobic interactions between polymer chains and the solvent (Hasirci and Hasirci, 2018). A change in temperature disrupts these interactions, leading to intra- and intermolecular interactions that result in the precipitation of the polymer, or in the case of a hydrogel, to a shrinkage or expansion (due to chain collapse and chain expansion, respectively). Polymer solutions that have an upper critical solution temperature (UCST) exhibit monophasic behavior above this temperature and undergo phase separation below UCST. Inversely, polymer solutions that have a lower critical solution temperature (LCST) undergo phase separation above this temperature and exhibit monophasic behavior below it. For example, a shrunken hydrogel with UCST of 25°C would swell and expand when introduced to the human body (37°C). These materials are widely utilized in drug delivery applications and tissue engineering applications, such as cell sheet engineering. Some examples are poly(*N-*isopropylacrylamide) (PNIPAM) (Ozturk et al., 2009), poly(*N*-vinylcaprolactam) (PNVC) (Haq et al., 2017), gelatin and GelMA (Kolesky et al., 2014), collagen and ColMA, methylcellulose, agarose, pluronic (Fedorovich et al., 2009), and poly(ethylene glycol) based block polymers (Suntornnond et al., 2017).

##### Photo responsive polymers

Photo responsive polymers undergo physical or chemical transformation upon exposure to light. Photo-stimulation can induce changes, such as conformation, polarity, hydrophilicity, charge, or bond strength which is translated to changes in the wettability, solubility, optical properties, and degradability of the material (Cabane et al., 2012). It has advantages, such as remote application with zero contact and ease of dose adjustment to control response strength (Cui and Del Campo, 2014). The photo responsiveness is due to presence of photosensitive side groups (chromophores) on the polymer backbone. Azobenzenes, spiropyrans, spirooxazines, diarylethenes, and fulgides are families of these side groups commonly found in photosensitive polymeric systems (Ercole et al., 2010). Depending on the type of chromophore present, the response induced can be reversible or irreversible. Irreversible photo responsive polymers are generally photodegradable polymers that are utilized in the development of drug delivery systems. For tissue engineering applications, 4D printing of hydrogels that swell or shrink or self-assemble upon photo-stimulation is an area waiting to be explored. Examples of such systems are poly(N-isopropylacrylamide) (PNIPAM) functionalized with spirobenzopyran (Sumaru et al., 2006), and a hydrogel system consisting of 4,4′-azodibenzoic acid (ADA), α-cyclodextrin, and dodecyl (C_12_)-modified poly(acrylic acid) (PAA) (Tomatsu et al., 2005).

##### Magneto-responsive polymers

Magneto-responsive polymeric systems are, in general, polymer networks physically or chemically functionalized with magnetic nanoparticles (MNP) which consist of magnetic elements, such as iron (Fe), cobalt (Co), nickel (Ni), or their oxides (Montero et al., 2019). When physically entrapped (by blending, *in situ* precipitation, or dip coating methods) or covalently immobilized, these magnetic nanoparticles introduce responsiveness to a magnetic field (Adedoyin and Ekenseair, 2018). This responsiveness results in a spatio-temporal control over the physical, structural, and mechanical properties of the polymeric scaffold. The degree and uniformity of the response depends on the types of the polymers and MNPs and their ratio, along with the distribution of MNPs within the matrix.

The potential of magneto-responsive materials in biomedical applications, has been demonstrated in many targeted drug delivery applications, where they offer minimally invasive, locally effective, and controlled therapeutic action (Chang et al., 2011; Peters et al., 2016; Casolaro and Casolaro, 2017). From a tissue engineering perspective, manipulations on the direction and strength of the magnetic field will result in specific alterations of scaffold morphology and geometry, and this can be used in certain tissue regeneration applications that require structural alignment (Xu et al., 2011; Panseri et al., 2012; Kokkinis et al., 2015), mechanical stimulation (Sapir-Lekhovitser et al., 2016), and stem cell differentiation (Fuhrer et al., 2013).

The disadvantage of using magnetic nanoparticles in living systems is that when leached from the matrix, MNPs smaller than 50 nm are able to cross biological membranes and adversely affect the function of the tissues by inducing inflammation, generating reactive oxygen species, impeding DNA function, and driving cells to apoptosis (Adedoyin and Ekenseair, 2018). Thus, the biocompatibility of any given magneto-responsive polymer is directly related to the type of MNPs used and the method of their incorporation into the polymeric network. Furthermore, the behavior of magneto-responsive materials under *in vivo* conditions should be estimated prior to application, using proper models that consider the magnetic field strength, the amount of MNPs used, and the responsiveness of these MNPs to the applied magnetic field, in order to achieve a controlled and successful therapeutic action (Pernal et al., 2018). These models would provide information on the appropriate manner and amount of magnetic stimulation required to induce tissue regeneration. This information is crucial, especially for vascular and osteochondral tissue applications where mechanotransduction plays an important role in induction of regeneration.

Examples of 3D printed polymeric magneto-responsive systems used in tissue engineering applications are iron(III)oxide (Fe_3_O_4_) nanoparticles containing mesoporous bioactive glass/poly(ε-caprolactone) (Fe_3_O_4_/MBG/PCL) (Zhang et al., 2014), magnetic nanocomposite scaffolds consisting of iron(III)oxide/PCL and iron(III)oxide/poly(ethylene glycol diacrylate) (PEGDA) (De Santis et al., 2015), and PCL/iron-doped hydroxyapatite (PCL/FeHA) nanocomposite scaffolds (D'Amora et al., 2017).

#### Responsiveness to Chemical Stimuli

##### pH responsive polymers

pH responsive polymers are polyelectrolytes that bear weak acidic or basic groups in their structure that accept or release protons in response to environmental pH changes. Carboxyl, pyridine, sulfonic, phosphate, and tertiary amine groups in polymers ionize with changes in pH which results in structural or property changes, such as solubility, degradability, configuration, chain conformation swelling, surface activity, and self-assembly (Reyes-Ortega, 2014). pH responsive polymer systems have been utilized in several biomedical applications, such as drug delivery (Bagherifam et al., 2015), gene delivery, and glucose sensors due to their unique properties.

In pH responsive systems, polymers of basic monomers act as cationic polymers under acidic conditions and polymers of acidic monomers behave as anionic polymers under basic conditions. One of the two types of polymers or a combination of the two can be implemented in a stimuli responsive system depending on the application. The origin of pH responsive polymers can be natural or synthetic. Poly(L-glutamic acid) (PGA), poly(histidine) (PHIS), poly(acrylic acid) (PAA) (Dutta and Cohn, 2017), and poly(aspartic acid) (PASA) are examples of synthetic pH responsive polymers which are biocompatible and biodegradable [except for poly(acrylic acid)], while chitosan, hyaluronic acid, gelatin, alginic acid, and dextran are examples of pH responsive polymers of natural origin (Kocak et al., 2017). pH differences are observed in many compartments of the human body (acidic environment in the stomach and basic environments of the intestines along the gastrointestinal tract, or the hypoxic nature of tumor tissue microenvironment), and the responsiveness of these materials can be exploited in tissue engineering applications.

##### Humidity responsive polymers

**Figure 7:**
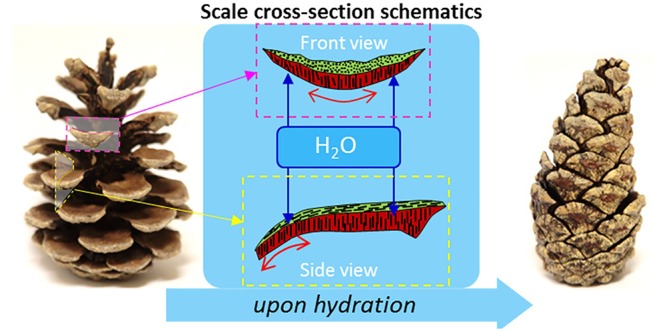
Stimulus responsiveness of pinecones. (Left) Dehydrated open pinecone; (Right) hydrated closed pinecone. Differences in the cellulose fibril winding angles in the top and bottom layers of the cells (sclereids in red and sclerenchyma in green) controls the expansion of these cells during hydration/dehydration. The cooperative anisotropic expansion of these differentiated cells results in the opening and closing of pinecone scales in response to hydration (adapted from Mulakkal et al., 2018).

Humidity responsiveness is a phenomenon that has many examples in nature. One such example is the movement of pinecone scales to preserve or dispense the seeds in response to the level of environmental humidity (Figure 7). The scales contract upon the increase of humidity and expand when the humidity level is low, scattering the seeds they hold inside (Song et al., 2015). These biological systems inspired the development of humidity responsive materials that release or absorb moisture in response to changes in humidity of the environment (Li Y. C. et al., 2016). Systems composed of these materials are able to transform the sorption or desorption of moisture into driving forces for movement. Poly(ethylene glycol) diacrylate (PEGDA) (Lv et al., 2018), cellulose (Mulakkal et al., 2018), and polyurethane copolymers (Jung et al., 2006) are some examples of humidity responsive materials that have been studied.

## 3D Printing Applications

Providing extreme control over the shape and architecture of the scaffolds makes 3D printing very attractive for the fabrication of the tissue engineering products. In this section, 3D printing applications for different types of tissues, such as bone, skin, nerve, vasculature, and other tissues are presented.

### Bone Tissue Engineering Applications

Bone is a mineralized tissue which has a high mechanical strength. Therefore, the mechanical properties of the printed polymers should be enhanced to match the properties of the bone tissue. There are many studies in the literature presenting the fabrication of 3D printed scaffolds for bone tissue engineering (Lee et al., 2011; Kao et al., 2015; Petrochenko et al., 2015; Saito et al., 2015; Wang et al., 2016). To strengthen the products, minerals, such as hydroxyapatite (HAP) and tricalcium phosphate (TCP) are blended with the polymers and then printed (Eosoly et al., 2012; Buyuksungur et al., 2017). Poly(ε-caprolactone) (PCL) is the most commonly used polymer for 3D printing of scaffold for bone tissue (Liao et al., 2011; Wang M. O. et al., 2015). PCL is a biodegradable, biocompatible and FDA approved polymer for certain medical applications. One of the main reasons for using this polymer is its relatively low T_g_ (~60°C) and T_m_ which makes it a very good compound for fused deposition modeling (Lee et al., 2016). However, PCL is a hydrophobic polymer and does not have any cell attractive moieties. In one report, it was blended with poly(propylene fumarate) (PPF) to increase the hydrophilicity of the 3D printed PCL scaffolds (Buyuksungur et al., 2017). Other materials, such as graphene and bioactive borate glass were also added for the production of composite scaffolds with PCL in order to improve the properties of the printed constructs (Wang et al., 2016; Murphy et al., 2017). Bone morphogenic protein (BMP) is another substance incorporated in 3D printed scaffolds either as is or in microparticles to increase the healing rate of the bone tissue, because a large number of studies showed the positive effect of BMPs on bone regeneration (Huang et al., 2005; Yilgor et al., 2009; Liu et al., 2013).

Different types of cells are used in bone tissue engineering applications. Among them, mesenchymal stem cells (MSCs) isolated from bone marrow or adipose tissue are the most frequently used ones (Duarte Campos et al., 2016; Cunniffe et al., 2017). Their high capacity to differentiate into bone cells makes them an ideal cell type to study and achieve bone regeneration. Some researchers added human umbilical vein endothelial cells (HUVECs) on the 3D printed scaffolds to achieve vascularization at the defect site (H. Cui et al., 2016). These cells were seeded to produce a tissue engineered bone tissue. However, recent studies focus on printing the cells together with the scaffold (Bendtsen et al., 2017; Keriquel et al., 2017; Wenz et al., 2017). For these applications, agarose, alginate, collagen, GelMA, methacrylated hyaluronic acid (HAMA), and PEG dimethacrylate (PEGDMA) hydrogels were used as bioinks. Nano HAP is also blended with these hydrogels in order to improve the mechanical properties of the printed constructs (Bendtsen et al., 2017; Cunniffe et al., 2017; Keriquel et al., 2017). MC3T3, which is an osteoblast precursor cell line was also commonly used in bioprinting applications (Lee et al., 2011; Eosoly et al., 2012).

Three-dimensional printed tissue engineered products were implanted *in vivo* at the defect site in order to study their effect on bone regeneration (Lee et al., 2011; Loozen et al., 2013; Saito et al., 2015; Buyuksungur et al., 2017; Keriquel et al., 2017). MSCs are incorporated with the PCL based scaffolds, and are reported to improve the bone regeneration when applied to rabbit femurs (Buyuksungur et al., 2017). Bone tissue engineering applications of 3D printing discussed here are summarized in Table 5.

**Table 5:** 3D printing of polymers for tissue engineering applications.

**Target tissue**	**Printing method**	**Printing material**	***In vitro* study**	***In vivo* study**	**References**
Bone	FDM	PCL, HAP, PPF	Rabbit bone marrow stem cells (BMSCs)	Femurs of rabbits	Buyuksungur et al., 2017
Bone	Continuous digital light processing	PPF	Angiogenesis modeling (representing endothelial cells)	Rat subcutaneous implantation	Wang M. O. et al., 2015
Bone	FDM	PCL	Pre-osteoblast MC3T3-E1	–	Lee et al., 2016
Bone	Extrusion based AM	PCL, graphene	Human adipose derived MSCs	–	Wang et al., 2016
Bone	SLS	PCL	Porcine adipose derived stem cells	–	Liao et al., 2011
Bone	SLS	PCL, HAP	Osteoblast-like cells MC 3T3	–	Eosoly et al., 2012
Bone	SLA	PPF/diethyl fumarate (DEF)	Pre-osteoblast MC3T3-E1	Cranial bone defect in rat	Lee et al., 2011
Bone	SLA	PLA coated with PDA	Human adipose derived stem cells	–	Kao et al., 2015
Bone	RP	PLLA, PCL	Human gingival fibroblasts	Subcutaneous implantation in mice	Saito et al., 2015
Bone	Two-photon polymerization	Urethane, acrylate based photo elastomer	Human BMSCs	–	Petrochenko et al., 2015
Bone	Bioprinting	PCL/bioactive borate glass	Human adipose stem cells	–	Murphy et al., 2017
Bone	Bioprinting	Alginate	Multipotent stromal cells	Subcutaneous implantation in nude mice	Loozen et al., 2013
Bone	Bioprinting	Alginate/PVA/HAP hydrogel	Mouse calvaria 3T3-E1 (MC3T3)	–	Bendtsen et al., 2017
Bone	LAB	Collagen, nano-HAP	Mouse MSCs	Calvaria defect model in mice	Keriquel et al., 2017
Bone	Bioprinting	PLGA, PEG	Immortalized human MSCs	–	Sawkins et al., 2015
Bone-Cartilage	Inkjet bioprinting	PEGDMA, GelMA	Human MSCs	–	Gao et al., 2015
Bone	Bioprinting	Collagen type I, agarose hydrogel	Human bone marrow derived MSCs	–	Duarte Campos et al., 2016
Bone	Bioprinting	Agarose hydrogel	3T3 murine embryonic fibroblasts	–	Carlier et al., 2016
Bone	Dual 3D bioprinting	PLA fibers, GelMA	hMSCs and HUVECs	–	Cui et al., 2016
Bone	Bioprinting/FDM	PCL, alginate and nano-HAP	Bone marrow derived MSCs	–	Cunniffe et al., 2017
Bone	Bioprinting	GelMA, HAMA, HAP	Human adipose derived stem cells	–	Wenz et al., 2017
Skin	Extrusion based printing	Silk sericin (SS), GelMA	L929, HaCaT and HSF cells	Mouse subcutaneous implantation	Chen et al., 2018
Skin	LAB	Collagen	NIH-3T3 and HaCaT	–	Koch et al., 2012
Skin	Extrusion based bioprinting	Chitosan, gelatin	HFF-1 cells	–	Ng et al., 2016
Skin	Free-form fabrication (FFF)	Fibrin	hFBs and hKCs	Immunodeficient athymic mice	Cubo et al., 2016
Skin	Bioprinting	Gelatin, alginate, fibrinogen	Human dermal fibroblasts (HDFs)	–	Pourchet et al., 2017
Skin	LAB	Matriderm^®^	Fibroblasts and keratinocytes	Dorsal skin fold chamber in nude mice	Michael et al., 2013
Skin	Extrusion and inkjet printing	Skin-derived extracellular matrix (S-dECM)	HDFs, human epidermal keratinocyte (HEK), human adipose derived MSCs, EPCs	Dorsal wound of BALB/cA-nu/nu mice	Kim et al., 2018
Nerve	Inkjet printing	Fibrin	Primary embryonic hippocampal, cortical neurons	–	Xu et al., 2006
Nerve	Direct inkjet printing	Collagen	Rat embryonic astrocytes, neurons	–	Lee et al., 2009
Nerve	Direct inkjet printing	Collagen, fibrin VEGF release	Murine neural stem cells (C17.2)	–	Lee et al., 2010
Nerve	Two-photon polymerization	Photopolymerizable PLA	SH-SY5Y human neuronal cell line, rat SCs	–	Koroleva et al., 2012
Nerve	Bioprinting	Agarose rods as supports, scaffold-free	Mouse BMSCs, SCs	Rat sciatic nerve injury model	Owens et al., 2013
Nerve	Piezoelectric inkjet printing	–	Adult rat retinal ganglion cells, retinal glia	–	Lorber et al., 2014
Nerve	Bioprinting	Gellan gum-RGD	Primary cortical neurons	–	Lozano et al., 2015
Nerve	FDM, bioprinting	Polyurethane	NSCs	Zebrafish embryo neural injury model	Hsieh et al., 2015
Nerve	Microextrusion bioprinting	Alginate, carboxymethyl-chitosan, agarose	Cortical human NSCs	–	Gu et al., 2016
Nerve	SLA-Low-level light therapy	GelMA and PEGDA	Mouse NSCs	–	Zhu et al., 2017
Nerve	SLA	PEGDA	NSCs	–	Lee S.-J. et al., 2017
Vascular	Digital light processing SLA	PPF	HUVECs, human umbilical vein SMCs	Mice animal model	Melchiorri et al., 2016
Vascular	E-jet 3D printing	PCL	HUVECs	Segment of the abdominal artery in rats	Huang et al., 2018
Vascular	Bioprinting	Multicellular spheroids, scaffold-free	HUVECs, HASMCs, human normal dermal fibroblasts (HNDFB)	Implantation in nude rats	Itoh et al., 2015
Vascular	RP bioprinting	Multicellular spheroids, scaffold-free	Smooth muscle cells, fibroblasts	–	Norotte et al., 2009
Cardiovascular	3D cell printing	MSCs-laden heart tissue-derived decellularized ECM	Human c-kit + cardiac progenitor cells (hCPCs)	Subcutaneous implantation in nude mice/rat myocardial infarction model	Jang et al., 2017
Vascular	Bioprinting	MEF cell aggregates	Mouse embryonic fibroblast (MEFs)	–	Kucukgul et al., 2015
Vascularization	Bioprinting	Matrigel/alginate	Endothelial progenitor cells (EPCs)	Subcutaneous implantation in nude mice	Poldervaart et al., 2014
Cartilage	SLS	PCL, collagen hydrogel	Chondrocytes	Dorsal area of 6-weeks-old male nude mice	Chen et al., 2014
Cartilage	Inkjet bioprinting	Nanocellulose, alginate	Human chondrocytes	–	Markstedt et al., 2015
Cartilage	Low-temperature FDM	Polyurethane	MSCs	Rabbit osteochondral defect	Hung et al., 2016
Cartilage	Electromagnetic jet technology	Nanofibrillated cellulose and alginate	Human nasal chondrocytes (hNC)	–	Martínez Ávila et al., 2016
Cartilage	Extrusion based bioprinting	Collagen, alginate, agarose	Primary rat chondrocytes	–	Yang et al., 2018
Meniscus	SLA	GelMA	Human avascular zone meniscus cells	Meniscus defect in an explant organ culture model	Grogan et al., 2013
Meniscus	FDM	PCL	–	–	Cengiz et al., 2016
Meniscus	FDM	PCL	–	–	Szojka et al., 2017
Meniscus	FDM	PCL	Porcine fibrochondrocytes	–	Bahcecioglu et al., 2018
Meniscus	FDM	PCL	Porcine fibrochondrocytes	–	Bahcecioglu et al., 2019
Cornea	Extrusion based bioprinting	Collagen, alginate	Corneal keratocytes	–	Isaacson et al., 2018
Cornea	LAB	Recombinant human laminin and collagen	Human ESC derived limbal epithelial stem cells, hASCs	Porcine organ culture	Sorkio et al., 2018
Urethra	Bioprinting	PCL, PLCL	Urothelial cells (UCs), SMCs	–	Zhang K. et al., 2017

### Skin Tissue Engineering Applications

Skin is a soft tissue; therefore, hydrogels are commonly used in 3D printing of skin substitutes. Many of the 3D printing technologies, such as extrusion-based printing and laser-assisted bioprinting are used to fabricate skin constructs (Koch et al., 2012). Tissue engineering is also used in the production of whole skin constructs to treat burns or chronic wounds. Collagen-based materials are used in most of the printing studies, as collagen is the main component of native skin. However, collagen has poor printability and a long crosslinking time (Ng et al., 2016). Chitosan is preferred over collagen for wound healing applications due to its antimicrobial properties and ability to trigger hemostasis. Alginate (Pourchet et al., 2017), gelatin (Ng et al., 2016), GelMA (Chen et al., 2018), and fibrin (Cubo et al., 2016) are also used to print skin constructs.

In most studies, bioprinting of skin grafts is achieved with the use of both hydrogels and skin cells (Vijayavenkataraman et al., 2016). Fibroblasts (NIH-3T3) and keratinocytes (HaCaT) are widely used because they are the main cell types in the skin tissue (Michael et al., 2013). Different types of skin cells should be placed in a skin mimicking organization within a 3D printed construct in order to create native human skin (Ng et al., 2015). Some of the 3D printed skin grafts were tested *in vivo* and achieved regeneration of the tissue at the injury site (Cubo et al., 2016; Kim et al., 2018). Skin tissue engineering applications of 3D printing are summarized in Table 5.

### Nerve Tissue Engineering Applications

Nerve tissue has a directional (uniaxial) organization due to the anisotropic orientation of the nerve fibers. Nerve guides are used to bring the proximal and the distal ends of a damaged nerve after an injury occurs. They can also be fabricated by 3D printing to provide patient-specific constructs with a complex inner architecture. Various types of additive manufacturing (AM), such as ink jet printing, stereolithography (SLA), fused deposition modeling (FDM) and bioprinting are frequently used in order to produce nerve tissue engineering products. Fibrin (Xu et al., 2006), collagen (Lee et al., 2009), PLA (Koroleva et al., 2012), gellan gum (Lozano et al., 2015), carboxymethyl chitosan (Gu et al., 2016), agarose (Owens et al., 2013), polyurethane (Hsieh et al., 2015), GelMA (Zhu et al., 2017) and PEGDA (Lee S.-J. et al., 2017) were also used to print constructs for nerve tissue engineering applications.

Mostly neural stem cells (NSCs) are incorporated into the constructs, however, glial cells, primary cortical neurons, astrocytes, Schwann cells (SCs), bone marrow stem cells (BMSCs), and retinal ganglion cells (RGCs) were also used in nerve regeneration studies (Zhu et al., 2017). The nervous system is composed of different types of cells, such as neurons, glial cells, and SCs, and therefore, many studies have concentrated on printing these cell combinations to obtain a whole nerve tissue construct (Koroleva et al., 2012). The orientation of these cells is also important because the nerve tissue is anisotropic. Bioprinting provides a very good solution to this problem since cells can be printed within hydrogel tubes or on fibers in a specific orientation, using this technique (Hsieh et al., 2015). Molecules of biochemical importance can also be added into the 3D printed structures. For example, vascular endothelial growth factor (VEGF) was incorporated into fibrin gel, and murine neural stem cells were shown to migrate toward this gel and exhibit an elongated shape with neurite-like extensions (Lee et al., 2010).

The number of *in vivo* studies is relatively few when compared with other tissues, mostly due to the complexity of the nervous system. In an *in vivo* study in rats, a mainly cellular nerve graft, composed of mouse bone marrow stem cells (BMSCs) and Schwann cells (SCs), was printed and tested in a sciatic nerve injury model (Owens et al., 2013). Tubular structures loaded with spheroids were deposited layer-by-layer into the agarose hydrogel. Extensive axonal regrowth across the biofabricated grafts was observed. In another study, 3D-printed NSC-loaded polyurethane (PU) constructs were tested in a zebra fish embryo neural injury model (Hsieh et al., 2015). After creating a defect in the nervous system, PU dispersions and NSCs were mixed, or only NSC suspensions were printed and implanted at the defect site. The adult zebra fish with a traumatic brain injury recovered after implantation of 3D printed NSC carrying PU constructs. Embryos injected with only NSCs showed low cell survival and the NSCs were not distributed in an aligned fashion.

3D printing was also combined with other techniques to enhance the properties of the nerve tissue constructs. For example, electrospinning together with printing was tested to increase the mechanical properties of the scaffolds (Lee S.-J. et al., 2017). PEGDA scaffolds were printed on electrospun PCL or PCL/gelatin fibers. Scaffolds with PCL/gelatin fibers had more neural stem cells that adhered, the average neurite length increased and directed neurite extension of primary cortical neurons was observed along the fibers. Nerve tissue engineering applications of 3D printing are also summarized in Table 5.

### Vascular Tissue Engineering Applications

Vascularization is one of the most important aspects of tissue regeneration, therefore, 3D printing introduces vascularization strategies and adds its advantages to create vasculature and therefore, healthy vascularized constructs (Duan, 2017). Even though stereolithography and ink-jet printing are used to print PCL and PPF products, most of the applications nowadays focus on bioprinting (Melchiorri et al., 2016; Huang et al., 2018). HUVECs are the most preferred cell type in cell printing studies to achieve vascularization (Itoh et al., 2015). Smooth muscle cells (SMCs) and fibroblasts are also incorporated into the construct structure. In some studies, scaffold-free constructs composed of multicellular aggregates, spheroids were printed (Norotte et al., 2009). Three-dimensional printed vascular grafts made of Matrigel and endothelial progenitor cells (EPCs) were tested on mice (Poldervaart et al., 2014). MSC-loaded heart tissue-derived ECM were also implanted in rats (Jang et al., 2017). All these studies showed promising vascularization results. Vascular tissue engineering applications of 3D printing are also summarized in Table 5.

### Other Tissue Engineering Applications

Three-dimensional printing has been employed in cartilage, meniscus, cornea, and urethra tissue engineering applications. For example, for cartilage tissue engineering, SLS (Chen et al., 2014), ink-jet bioprinting (Markstedt et al., 2015), and extrusion-based bioprinting (Yang et al., 2018) were used. Nanocellulose, alginate, polyurethane (PU), collagen, and agarose are used as the printing polymers. Chondrocytes are the most frequently used cells in cartilage regeneration (Martínez Ávila et al., 2016). In a study, a bioactive molecule, TGF β3, was incorporated into 3D printed PU constructs to achieve cartilage regeneration (Hung et al., 2016). The scaffolds promoted self-aggregation of MSCs with a controlled release of the bioactive ingredients and when implanted into rabbit osteochondral defects, they showed cartilage regeneration at the defect site.

Three-dimensional printing techniques are also used in meniscus tissue engineering applications. In a study, GelMA scaffolds printed with SLA were implanted into a meniscus defect in an explant organ culture model (Grogan et al., 2013). Results demonstrated that micropatterned GelMA scaffolds produce cellular alignment and promoted meniscus-like tissue formation. PCL is one of the most commonly used polymers for cartilage tissue engineering applications (Cengiz et al., 2016; Szojka et al., 2017). An artificial meniscus shaped PCL scaffold was printed with cartilage-like inside and fibrocartilage-like outer component (Bahcecioglu et al., 2018). In that study, agarose (Ag) and GelMA hydrogels were added onto PCL as the inner and outer regions, respectively. Ag increased glycosaminoglycan (GAG) production 4-fold, while GelMA enhanced collagen production ca. 50-fold after being seeded with porcine fibrochondrocytes. In a related study, porcine fibrochondrocyte-seeded hydrogels, such as agarose, GelMA, HAMA, and GelMA-HAMA were combined with 3D printed PCL scaffolds and evaluated under static and dynamic compression conditions (Bahcecioglu et al., 2019). After 35 days, cell carrying hydrogels produced higher levels of ECM components than the 3D printed PCL control.

A limited numbers of studies were reported for 3D printing of corneal tissues. Collagen-based 3D bioprinted scaffolds containing corneal keratocytes were studied and keratocytes exhibited high cell viability on days 1 (>90%) and 7 (83%) in the culture medium (Isaacson et al., 2018). In a different study, 3D cornea mimicking tissues were constructed by laser assisted bioprinting (LAB) using human embryonic stem cell derived limbal epithelial stem cells (hESC-LESC) and human adipose tissue derived stem cells (hADSCs) (Sorkio et al., 2018). The structure of the 3D bioprinted stroma showed that the hADSCs aligned horizontally and also demonstrated expression of collagen type I. They attached to the host tissue with hADSCs migration from the printed structure after 7 days in porcine organ cultures.

Zhang et al. printed cell-loaded urethra in order to mimic the structure and mechanical properties of the natural urethra of rabbits (Zhang K. et al., 2017). The tubular scaffold was fabricated using an integrated bioprinting system, with urothelial cells (UCs) and smooth muscle cells (SMCs). Results showed that mechanical properties of the polycaprolactone (PCL)/poly(lactide-co-caprolactone) (PLCL) (50:50) spiral scaffold were equivalent to the native urethra in the rabbit. Both UCs and SMCs maintained more than 80% viability 7 days after printing and expressed specific biomarkers in the cell-loaded hydrogel.

In some studies, 3D printing techniques are combined with near field electrospinning (NFES) to introduce highly aligned and reproducible fibrous structures into the 3D printed scaffolds. NFES technology provides precise control over the orientation of the fibers. Therefore, it is generally used in the development of anisotropic tissues, such as the nerve, cornea, and muscle (He et al., 2017). In this study, melt near field electrospinning was used in a direct writing mode onto a rotating cylindrical collector (drum) to fabricate tubular scaffolds (Brown et al., 2012). Primary human osteoblasts (hOB), mouse osteoblasts (mOB), and human mesothelial cells infiltrated into the fibrillar scaffolds, and the resultant architecture produced by the application of these processes was found to be supportive of cells spanning between adjacent fibers. Yan et al. (2014) also printed chitosan-gelatin composite scaffolds, and chitosan-PVA fibers produced by NFES were integrated with this 3D printed structure. This macro/micro-controlled tissue engineering scaffold had proper porosity (55%) and mechanical strength (modulus of elasticity of 288 MPa). In another study, poly(methyl methacrylate) (PMMA) fibers were printed in between 3D collagen gels loaded with hMSCs to create an anisotropic platform for cell growth and proliferation (Fattahi et al., 2017). Aligned PMMA fibers supported hMSCs growth, aligned them within the gels, and increased the anisotropic properties of gels.

## 4D Printing Applications

Four-dimensional printing includes groups of programmable responsive self-assembly, self-folding or self-accommodating technologies (An et al., 2016). Programmable design, the 3D printing process, and triggering by external stimuli, such as temperature and light are the three main components of 4D printing. Smart materials which have the ability to change their properties under the influence of external signals are the basis of 4D printing applications (Khoo et al., 2015).

Like in 3D printing applications, SLA (Raman et al., 2016), AM (Hendrikson et al., 2017), FDM (Miao et al., 2016b), and bioprinting (Pati et al., 2015) techniques are employed in 4D printing applications. Four-dimensional bioprinting is used in tissue engineering applications because it is possible to fabricate sensitive and complex structures by 4D printing (Gao et al., 2016). Responsive materials, such as poly(N-isopropylacrylamide-co-acrylic acid) (pNIPAM-AAc) (Breger et al., 2015), methacrylated polycaprolactone (Zarek et al., 2016b), polycaprolactone triol (Miao et al., 2016a), nanofibrillated cellulose (Gladman et al., 2016), soybean oil epoxidized acrylate (Miao et al., 2018), iron(III)oxide (Fe_3_O_4_) nanoparticles containing mesoporous bioactive glass/poly(ε-caprolactone) (Fe_3_O_4_/MBG/PCL) (Zhang et al., 2014), magnetic nanocomposite scaffolds consisting of PCL/Fe_3_O_4_ and poly(ethylene glycol diacrylate) (PEGDA)/Fe_3_O_4_ (De Santis et al., 2015), and PCL/iron-doped hydroxyapatite (PCL/FeHA) nanocomposite scaffolds (D'Amora et al., 2017) are used as printing materials for 4D printing. They respond to temperature, light, magnetic field, humidity, and change their properties, but mainly the shape. Mostly mesenchymal stem cells (MSCs) are utilized in *in vitro* studies of the 4D printed scaffolds (Pati et al., 2015). There are limited number of *in vivo* studies since it is a relatively new technique. Tissue engineering applications of 4D printing are summarized in Table 6.

**Table 6:** 4D printing of polymers for tissue engineering applications.

**Application**	**Technique**	**External stimulus**	**Polymer type**	**Cells used**	**References**
Fabrication of 3D tissue constructs	Extrusion based bioprinting	Biological (Cell-laid mineralized ECM)	PCL, PLGA, β-TCP	Human nasal inferior turbinate tissue derived MSCs	Pati et al., 2015
Materials for self-evolving deformation	Inkjet printing	Humidity	Vinyl Caprolactam, Polyethylene	–	Raviv et al., 2014
Tissue engineering	Extrusion based AM	Humidity	Nanofibrillated cellulose	–	Gladman et al., 2016
Optogenetic muscle ring-powered biobots	SLA	Light	PEGDA	C2C12 murine myoblasts	Raman et al., 2016
Bone tissue engineering	FDM	Magnetic	Fe_3_O_4_/MBG/PCL	Human BMSCs	Zhang et al., 2014
Tissue engineering scaffolds	FDM and SLA	Magnetic	PCL/Fe_3_O_4_	Human MSCs	De Santis et al., 2015
			PEGDA/Fe_3_O_4_		
Bone tissue engineering	FDM	Magnetic	PCL/iron-doped HAP	Human MSCs	D'Amora et al., 2017
Endoluminal medical devices	UV-LED SLA	Temperature	Methacrylated polycaprolactone	–	Zarek et al., 2016b
Biomedical scaffolds	SLA	Temperature	Soybean oil epoxidized acrylate	Human bone marrow MSCs	Miao et al., 2016b
Tissue engineering scaffolds	FDM	Temperature	Polycaprolactone triol	Primary human bone marrow MSCs	Miao et al., 2016a
Cardiac regeneration	Photolithographic SLA-tandem strategy	Temperature	Soybean oil epoxidized acrylate	hMSCs	Miao et al., 2018
Soft robotic and surgical application	Photolithography	Temperature and Magnetic	Poly(*N*-isopropylacrylamide-*co*-acrylic acid)	L929	Breger et al., 2015

## Conclusion and Future Perspectives

Three-dimensional printing is becoming an indispensable tool in the production of devices and systems in biomaterials and tissue engineering areas. It changed the face of the biomaterials world with the production of patient specific devices that have the required shape and organization. Stimuli responsive materials, such as metals and polymers have been in use in the biomedical field, and the combination of material and responsiveness in a biomedical device creates 4D printing which introduces highly useful, viable, dynamic, and responsive systems in tissue engineering applications. As it is, 3D and 4D printing methods is still keeping researchers busy in their quest for producing novel biomaterials and biomedical devices. The current types of stimuli to which the materials are responsive to are quite well-known but is, unfortunately, limited. So, the development of different materials with multi-sensitivities for use in the enhancement of the dynamic nature of devices is still a challenging issue.

## Author Contributions

DT, TD, and AA wrote the first draft. DY, NH, and VH edited, revised, and finalized the text.

### Conflict of Interest Statement

The authors declare that the research was conducted in the absence of any commercial or financial relationships that could be construed as a potential conflict of interest.
